# A cross-sectional survey of knowledge, attitudes, and practices regarding antimicrobial resistance among Syrian health care professionals

**DOI:** 10.1016/j.ijregi.2025.100789

**Published:** 2025-10-19

**Authors:** Hisham Alhosni, Fares Alahdab, Wasim Zakaria, Ahmed Kejah, Mosa Shibani, Mohammad Basheer Alameer, Angie Hawat, André Torbey, Nabil Karah, Abduljabbar Alhajmousa, Abdulkarim Ezkazyez, Aula Abbara

**Affiliations:** 1 School of Public Health, Faculty of Medicine, Imperial College London, London, UK; 2 Department of Biomedical Informatics, Biostatistics, and Medical Epidemiology, University of Missouri, Columbia, USA; 3 Department of Clinical and Health Informatics, University of Texas Health Science Center at Houston, Houston, USA; 4 Faculty of Medicine, University of Aleppo, Aleppo, Syria; 5 Department of Pathology, University of Aleppo, Aleppo, Syria; 6 School of Health and Wellbeing, University of Glasgow, Glasgow, UK; 7 Faculty of Medicine, Damascus University, Damascus, Syria; 8 Department of General Surgery, Glasgow Royal Infirmary, Glasgow, UK; 9 Department of Molecular Biology and Umeå Centre for Microbial Research (UCMR), Umeå University, Umeå, Sweden; 10 R4HSSS Program, King’s College, London, UK; 11 King’s College London, London, UK; 12 Syria Public Health Network, London, UK; 13 Department of Infectious Disease, Imperial College London, London, UK

**Keywords:** Antimicrobial resistance, Antimicrobial stewardship, Syria

## Abstract

**Objectives:**

Antimicrobial resistance (AMR) poses a global challenge, especially in conflict-affected settings such as Syria, where health care infrastructure is compromised. This study assessed Syrian clinicians’ knowledge, attitudes, and practices regarding AMR and antimicrobial stewardship (AMS).

**Methods:**

A two-phase online survey was conducted from June-July to October-November 2024 among physicians, pharmacists, dentists, and nurses across 11 Syrian governorates. Participants were recruited through professional networks, social media, and snowball sampling.

**Results:**

Of 1179 respondents, 54% were male and 56% were residents (doctors in training); 32% had over 5 years’ experience. Most practiced medicine (78%), pharmacy (13%), or dentistry (8%). Awareness of AMR as a global (98%), national (93%), and local (67%) issue was high. Although 90% supported AMS, only 47% rated their hospital’s program as effective. Most desired more education (94%) and favored local guidelines (83%). Prescribing confidence was strong for routine cases (63%), but lower for complex ones (40-45%). Concerns included drug quality (80%), cost (52%), and ineffectiveness (47%). Notably, 39% struggled to withhold antibiotics in viral cases.

**Conclusion:**

The survey reveals strong AMR awareness but significant gaps in AMS practice, microbiology access, and clinical guidance. Strengthening Syria’s national AMR strategy is urgently needed.

## Highlights

• A survey of knowledge, attitudes, and practices regarding antimicrobial resistance and antimicrobial stewardship among 1179 participants indicates high awareness.
• Antimicrobial stewardship was supported by 90% of respondents, but only 47% reported having an effective program at their hospitals.
• Most desired more education (94%) and favored local guidance (83%).
• Concerns about drug quality (80%), cost (52%), and ineffectiveness (47%) exist.
• Addressing system-wide and local issues through multiple actions is urgently needed.

## Introduction

Overuse and misuse of antimicrobials are important drivers of antimicrobial resistance (AMR), a consequence that is also associated with health systems weakened by conflict. In conflict-affected Syria, the conflict has amplified the factors driving AMR, with widespread destruction of health care infrastructure, the exodus of health care workers, and a breakdown of infection control and antimicrobial stewardship (AMS) [1,2]. Forced displacement, resource scarcity, and a surge in injuries further exacerbated the situation [[1], [2], [3]]. Antibiotic shortages and interrupted supply chains, along with substandard antibiotic quality, misuse, and the absence of microbiology services, have all contributed to work-arounds, incorrect antibiotic use, and worsening of the AMR crisis [[2], [3], [4]]. Although legislation limiting over-the-counter dispensing of antibiotics without prescription existed before the conflict (law 2/T, 1992), compliance monitoring and enforcement mechanisms have been lacking [5,6].

In Syria, the prolonged conflict, the COVID-19 pandemic, the February 2023 earthquakes, and economic collapse with massive inflation have all contributed to the collapse of the health system and the exodus or death of thousands of health care professionals. These events have compounded pressures on the health system and remaining personnel [[7], [8], [9]] and have also exacerbated existing pre-conflict factors related to antibiotic overuse. Before the conflict, several studies showed that the rising irrational prescription practices, dispensation, and misuse of antibiotics were contributing to the rise of AMR and infections [10,11]. A 2010 study conducted in the Kalamoon region found that 57% of 365 participants purchased antibiotics without a prescription and lacked awareness of the associated risks [12]. This has been described in other studies in which physicians and pharmacists may overprescribe antibiotics in response to patient pressure, contributing to AMR [11,13,14]. This may be associated with concerns that their reputation or business may be affected if they decline prescriptions [7,[15], [16], [17]].

Despite the recognized challenges of AMR and AMS in Syria, there remains limited exploration of the drivers. In particular, the knowledge, attitudes, and practices (KAP) around AMR and AMS have not been explored. The aim of this cross-sectional survey was to investigate the KAP regarding AMR and AMS among health care professionals who prescribe or dispense antibiotics in different Syrian governorates.

## Materials and methods

### Ethical consideration

Ethical approval for this study was obtained from the Imperial College Research Ethics Committee (ICREC) (Supplementary File 1) on June 5, 2024, and from the Research Governance and Integrity Team (RGIT) on May 23, 2024. Under the ICREC process, the study was granted RGIT approval without requiring a full committee review, as no significant ethical issues were identified in the protocol or ethics application (ICREC Reference number: 7060010). Informed consent was obtained from all participants through the first part of the online questionnaire. Participant confidentiality was maintained by anonymizing the data and securely storing it to prevent unauthorized access. The study was conducted and reported in accordance with the STROBE (STrengthening the Reporting of Observational studies in Epidemiology) reporting guideline [18].

### Study design and setting

This cross-sectional KAP survey on AMR was introduced in two phases: the first, conducted in June-July 2024, mostly covered areas under former regime control, and the second, conducted in October-November 2024 in northern Syria, reached the remaining governorates. The reason for this was related to the geopolitical situation at the time (before the fall of the regime in December 2024) and the obtained approvals. The survey instrument remained identical across both phases, and the dissemination plan and data collection modality (online survey in Arabic) were similar.

### Study population

The target population was practicing and in-training health care professionals (physicians, pharmacists, and dentists). Inclusion criteria required participants to be aged ≥18 years and to provide informed consent. Exclusion criteria included residing and/or working outside Syria. The survey was conducted in Arabic, with instructions in plain language to ensure comprehension.

### Survey instrument

A structured questionnaire was developed to assess KAP regarding AMR. Initial items were drafted on the basis of published KAP studies related to AMR and guidelines by the World Health Organization. The questionnaire covered multiple domains, including (1) demographics (age, sex, governorate, specialty); (2) knowledge of antibiotic indications, dosing, and microbial resistance; (3) attitudes toward antibiotic stewardship and AMR-related concerns; and (4) reported practices in prescribing or recommending antibiotics. Supplementary File 2 includes the survey instrument.

Before full administration, the survey underwent pilot testing with a small sample (n = 10) of students and health care professionals to evaluate clarity, relevance, and approximate completion time. Minor revisions were made to wording and format on the basis of pilot feedback. The response formats included 5-point Likert scales and multiple-choice questions. Data were collected online. No follow-up procedures were conducted for non-respondents, as participation was entirely voluntary and based on a convenience sample.

### Data collection and survey administration

An electronic questionnaire (administered using Google Forms) was disseminated via social media platforms, email lists, and professional networks targeting the medical, dental, and pharmacy communities. A cover page explained the study objectives, voluntary participation, and confidentiality. Convenience and snowball sampling were used.

### Power analysis

A power analysis was conducted to determine the minimum sample size required for statistical significance. The analysis used an alpha level (α) of 5% (probability of a type I error), a power level of 0.80, and a conservative assumption that 50% of participants would have sufficient AMS knowledge. On the basis of these parameters, the minimum required sample size was calculated to be 614 participants.

### Statistical analysis

All statistical analyses were performed using R (R Core Team, 2025). Descriptive statistics were generated for demographic variables and key survey items. Means (± standard deviations) or medians (interquartile ranges) were calculated for continuous variables, depending on normality. Categorical variables were summarized as frequencies and percentages.

Analysis of variance was applied to compare mean confidence scores across categorical variables such as the professional role (e.g., student, resident, attending physician) or graduate specialties. Independent-samples *t*-tests were used to compare mean scores between two groups (e.g., male vs female participants). For variables violating normality assumptions, Kruskal-Wallis or Wilcoxon rank-sum tests were considered. Chi-square or Fisher’s exact tests were used, as appropriate, for categorical outcomes or associations (e.g., frequency of antibiotic prescribing). We also modeled prescribing frequency (an ordered five-level outcome from “less than once a week” to “more than once daily”) using a cumulative‐link (proportional-odds) ordinal logistic regression. Statistical significance was set at *P* <0.05. For *P*-values below 0.001, results were reported as “*P* <0.001.” Participants with significant missing demographic data or significant incomplete survey responses were excluded from analyses of the relevant variables. The number of excluded responses was documented (n = 6), and no imputation methods were used.

## Results

### Study participants

Among the 1179 participants, 54.2% (n = 649) were male. The main represented governorates were as follows: Idlib (29.6%), Aleppo (22.7%), and Damascus (14.1%). Respondents were predominantly physicians (77.6%, n = 915), with pharmacists (12.5%, n = 148), dentists (7.9%, n = 93), nurses (0.9%, n = 10), and midwives (0.7%, n = 8) comprising the remainder. In terms of career stage, 55.6% (n = 656) were residents, 14.8% (n = 175) attending physicians, and 21.3% (n = 251) allied professionals (dentists, pharmacists, or nurses). Practice experience skewed toward early career: 24.2% (285) had ≤1 year, 43.5% (n = 513) had 2-5 years (the median category), 13.3% (n = 157) had 5-10 years, and 10.5% (n = 124) had >10 years. Missing data were minimal for most variables, and Table 1 summarizes the demographics and other details of the study participants.

**Table 1:** Demographics of the survey participants.

		N (%)
Total N of participants		1179 (100)
Sex	Female	540 (45.8)
	Male	639 (54.2)
Governorate	Damascus	166 (14.08)
	Aleppo	268 (22.73)
	Daraa	31 (2.63)
	Hama	59 (5)
	Homs	81 (6.87)
	Idlib	349 (29.6)
	Lattakia	84 (7.12)
	Quneitra	9 (0.76)
	Rural Damascus	53 (4.5)
	Suwayda	30 (2.54)
	Tartus	43 (3.65)
	Missing	6 (0.51)
Branch	Medicine	915 (77.6)
	Pharmacy	148 (12.5)
	Nursing	10 (0.9)
	Obstetrics and gynecology	8 (0.7)
	Dentistry	93 (7.88)
	Missing	5 (0.42)
Graduate seniority	Resident	656 (55.6)
	Attending physician	175 (14.8)
	Dentistry, pharmacy, nursing	251 (15.6)
Experience	≤1 year	285
	2-5 years	513
	5-10 years	157
	>10 years	124
	Missing	55

### Confidence in AMS

Respondents generally felt confident in core AMS tasks, although confidence varied by activity (Table 2a). For general antibiotic dosing, 62.6% (n = 738) reported feeling confident “always” or “most of the time,” while 59.6% (n = 702) reported confidence in deciding treatment duration and 57.2% (n = 674) in microbiology-guided antibiotic selection (Table 2a). By contrast, only 40.2% (n = 474) felt highly confident when dosing for special cases and 45.2% (n = 533) when adjusting the antibiotic regimen if the patient’s condition did not improve. When initiating therapy, 93.1% (n = 1097) reported being “very” or “somewhat” confident (Table 2b).

**Table 2a:** Perceptions and knowledge of antimicrobials and their prescription.

	Always	Most of the time	Half the time	Sometimes	Rarely	Never
Confidence in general antibiotic dosing	130 (11%)	608 (51.6%)	311 (26.4%)	43 (3.6%)	44 (3.7%)	40 (3.4%)
Confidence in dosing for special cases, e.g., renal or liver failure	121 (10.3%)	353 (29.9%)	347 (29.5%)	61 (5.2%)	168 (14.2%)	126 (10.7%)
Confidence in adjusting antibiotics	99 (8.4%)	434 (36.8%)	386 (32.7%)	57 (4.8%)	137 (11.6%)	61 (5.2%)
Confidence in deciding treatment duration	154 (13.1%)	548 (46.5%)	326 (27.7%)	36 (3.1%)	77 (6.5%)	35 (3%)
Confidence in microbiology and antibiotic selection	172 (14.6%)	502 (42.6%)	279 (23.7%)	55 (4.7%)	100 (8.5%)	69 (5.9%)

**Table 2b:** Perceptions and knowledge of antimicrobials and antimicrobial resistance.

	Response	N (%)
Importance of antibiotic knowledge	No	5 (0.4%)
	Yes	1170 (99.2%)
	Missing	4 (0.3%)
Antimicrobial resistance is a problem	I don’t know	2 (0.2%)
	No	12 (1%)
	Yes	1153 (97.8%)
Confidence in starting antibiotics	Somewhat confident	754 (64%)
	Somewhat not confident	73 (6.2%)
	Very confident	343 (29.1%)
	Very not confident	8 (0.7%)
	Missing	1 (0.1%)

When stratifying by sex, male participants reported higher mean confidence scores than female participants for every task (general dosing, dosing special cases, adjusting regimens, microbiology-based selection, and deciding treatment duration; *P*-value <0.001) (Supplementary File 3: Figure 1). Confidence also varied by seniority and specialty (Supplementary File 3: Figure 2). The specialty-stratified plot (Supplementary File 3: Figure 2) reveals a wide variation in self-rated AMS confidence that corresponds to each group’s typical exposure to infectious disease management. Mean confidence scores in antibiotic selection were highest among the pulmonary, laboratory medicine, and rheumatology physicians, while confidence in deciding treatment duration was highest among neurologists and pulmonary care physicians.

**Figure 1:**
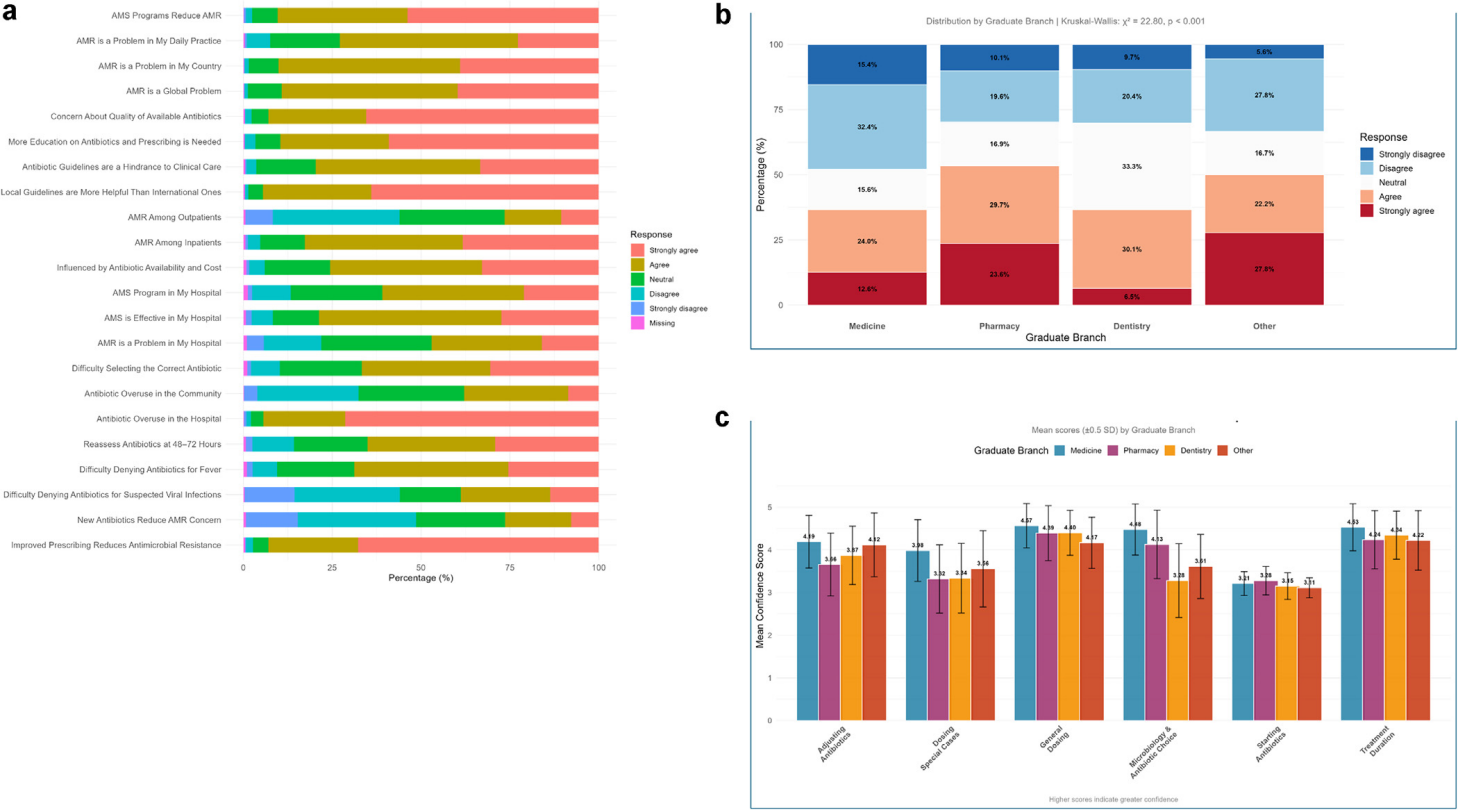
(a). Attitudes toward AMR and AMS. (b) Difficulty denying antibiotics for viral infections by cadre, including medicine (n = 913), pharmacy (n = 148), dentistry (n = 93), and other (n = 18). (c) Confidence levels across all antibiotic stewardship domains. AMR, antimicrobial resistance; AMS, antimicrobial stewardship.

### Knowledge and attitudes toward AMR and AMS

Responses showed near-universal recognition of AMR as a serious issue: 89.5% of participants (n = 1056) agreed or strongly agreed that AMR is a global problem, 93.0% (n = 1096) reported that it poses a threat in Syria, and 66.6% (n = 786) viewed it as a problem in their own hospital (Supplementary File 3: Table 1). After adjusting for sex, specialty, seniority, and governorate, attitudes were consistent between male and female participants for all of the aforementioned “AMR is a problem” statements. The only discrepancies noted for the “AMR is a global problem” statement were related to seniority: attendings and residents showed higher odds of agreeing with this statement than the students, with odds ratios (ORs) of 3.46 (95% confidence interval [CI] 1.35-8.88, *P* = 0.01) and 3.52 (95% CI 1.53-8.1, *P* = 0.003), respectively. By governorate, lower agreement with the “AMR is a problem in my hospital” statement was found in Idlib (OR 0.38 [95% CI 0.22-0.64], *P* <0.001), Lattakia (OR 0.43 [95% CI 0.24-0.77], *P* = 0.004), Rif Dimashq (OR 0.39 [95% CI 0.2-0.77], *P* = 0.006), and Tartus (OR 0.43 [95% CI 0.21-0.9], *P* = 0.024), when compared with Damascus.

Participants voiced several concerns about antibiotic quality and prescribing factors: 79.6% (n = 939) were worried about the quality of available antimicrobials, 71.2% (n = 840) about emerging resistance, 51.6% (n = 608) about costs, and 46.6% (n = 549) about ineffectiveness. Only 9.8% (n = 115) expressed concern about *Clostridium difficile*. Decisions were strongly influenced by availability and cost for 78.7% (n = 928), and 37.9% (n = 446) reported difficulty in selecting the correct antibiotic. When faced with viral infections, 38.9% (n = 458) found it difficult to withhold antibiotics, with similar patterns across sex, specialty, and governorate. However, students found it more difficult than attendings (OR 0.5, 95% CI 0.26-0.98, *P* = 0.045) or residents (OR 0.55, 95% CI 0.3-1.03, *P* = 0.063) to withhold antibiotics for viral infections (Supplementary File 3: Table 1).

Attitudes toward AMS were predominantly favorable: 90.1% (n = 1063) agreed that AMS programs improve patient care and safety, and 89.2% (n = 1052) agreed that they reduce AMR. However, only 46.9% (n = 554) judged AMS as effective in their own hospital, while 31.1% (n = 367) were neutral and 20.8% (n = 246) disagreed. A large majority (94.4%, n = 1114) considered that more education on antibiotic prescribing is needed, and 82.7% (n = 975) found local guidelines more helpful than international ones (although 26.4% [n = 312] viewed guidelines as a hindrance) (Figure 1a).

Access to training and resources was limited: 647 (54.9%) did not receive any training on AMR or AMS in the preceding year, and 692 (58.7%) did not have access to relevant continuous medical education courses. Regarding resources to support antibiotic prescriptions, usefulness ratings varied, with UpToDate and senior colleagues/specialists deemed very useful/useful by 684 (58%) and 638 (54.1%) participants, respectively (Table 3).

**Table 3:** Access to training and resources.

Frequency of having received training on antimicrobial resistance/antimicrobial stewardship in the past year:							
	Never	1-3 times	4-6 times	6-10 times	≥10 times	Missing	
Academic department activities	647 (54.9%)	425 (36%)	62 (5.3%)	30 (2.5%)	9 (0.8%)	5 (0.4%)	
Continuous medical education courses/professional development activities	692 (58.7%)	385 (32.7%)	61 (5.2%)	21 (1.8%)	11 (0.9%)	8 (0.7%)	

The rated usefulness of resources when needing help regarding antibiotic prescriptions:							
	Very useful	Useful	Somewhat useful	Not very useful	Not useful at all	Never used	Missing
Senior colleagues/specialists	357 (30.3%)	281 (23.8%)	330 (28%)	110 (9.3%)	48 (4.1%)	49 (4.2%)	4 (0.3%)
Peers	141 (12%)	234 (19.8%)	460 (39%)	177 (15%)	69 (5.9%)	91 (7.7%)	7 (0.6%)
UpToDate	486 (41.2%)	198 (16.8%)	141 (12%)	87 (7.4%)	70 (5.9%)	189 (16%)	8 (0.7%)
Sanford antimicrobial guide	235 (19.9%)	136 (11.5%)	107 (9.1%)	82 (7%)	40 (3.4%)	566 (48%)	13 (1.1%)
Local/national guidelines	178 (15.1%)	191 (16.2%)	234 (19.8%)	114 (9.7%)	62 (5.3%)	390 (33.1%)	10 (0.8%)

Availability of sufficient antimicrobial prescribing resources:							
	Yes	No	Missing				
	731 (62%)	441 (37.4%)	7 (0.6%)				
Availability of culture results:							
Missing	Available and trustworthy	Available but difficult to obtain	Available but expensive	Available but takes too long	Available but not trustworthy	Unavailable	
7 (0.6%)	224 (19%)	254 (21.5%)	416 (35.3%)	38 (3.2%)	146 (12.4%)	94 (8%)	

### Analysis by cadre

Additional analyses by cadre examined confidence in withholding antibiotics for viral infections and different AMS-related confidence parameters. Figure 1b shows the proportions of different cadres reporting difficulty in denying antibiotics to patients. There was a significant difference between medics and pharmacists (*P* = 0.000): 36.6% of doctors and 53.3% of pharmacists agreed or strongly agreed that they had difficulty denying antibiotics for viral infections. Figure 1c demonstrates no significant differences in confidence across different cadres and AMS domains. Doctors had the highest mean confidence scores in most domains, including antibiotic adjustment, dosing for special cases, general dosing, microbiology-based selection, and treatment duration; mean confidence in starting antibiotic therapy was similar across all cadres.

### Practices related to AMR

Prescribing frequency varied, with 39.8% (n = 469) of respondents reporting prescribing antibiotics more than once daily, and 21.1% (n = 249) reporting 3-5 times per week (Table 4). However, there was some variation in the frequency of prescribing across strata according to the multivariable logistic regression model, which adjusted for sex, seniority, specialty, and governorate. Men had 48% higher odds than women of reporting more frequent antibiotic prescribing (OR 1.48, 95% CI 1.17-1.88, *P* = 0.001). Respondents from Idlib had more than five times the odds of higher prescribing frequency compared with those in Damascus (OR 5.23, 95% CI 3.37-8.10, *P* <0.001); those from Rif Dimashq had approximately three times higher odds (OR 3.31, 95% CI 1.83-5.99, *P* <0.001), and those from Tartus had twice the odds (OR 2.36, 95% CI 1.25-4.45, *P* = 0.008). Certain specialties also exhibited a greater likelihood of frequent antibiotic prescribing, including pediatrics (OR 5.36, 95% CI 2.6-11.06, *P* <0.001), surgery (OR 4.02, 95% CI 2.1-7.67, *P* <0.001), obstetrics and gynecology (OR 3.97, 95% CI 1.89-8.34, *P* <0.001), and emergency medicine (OR 2.71, 95% CI 1.47-5.01, *P* = 0.001) (Supplementary File 3: Table 3).

**Table 4:** Clinical scenarios to test practices regarding antibiotic selection and spectrum.

(a) “What is the preferred antibiotic choice for a UTI (urinary tract infection) with or without pyelonephritis in a young woman who is not pregnant and has not had a previous hospital visit (Choose one)?” This tests knowledge about prescribing for urinary tract infections. Preferred answers are shown in gray, although choices would also depend on local resistance patterns, including extended-spectrum beta-lactamase rates.		
	Preferred antibiotic for UTI without pyelonephritis	Preferred antibiotic for UTI with pyelonephritis
Amoxicillin/clavulanic acid	117 (10.0%)	90 (7.7%)
Ceftriaxone	103 (8.7%)	345 (29.3%)
Ciprofloxacin	324 (27.5%)	442 (37.5%)
Meropenem	28 (2.4%)	140 (11.9%)
Nitrofurantoin	594 (50.4%)	147 (12.5%)
Missing	13 (1.1%)	15 (1.3%)
UTI, urinary tract infection.		

(b) “What are the most appropriate antibiotics for *Pseudomonas* sp. or anaerobic bacterial infection (choose 3)?” This tests knowledge of the antibiotic spectrum. Correct choices are shown in gray shading.		
	A *Pseudomonas* infection	An anaerobic bacterial infection
Ciprofloxacin	381	182
Meropenem	589	341
Piperacillin-tazobactam	202	444
Trimethoprim-sulfamethoxazole	278	-
Doxycycline	53	-
Amoxicillin-clavulanic acid	78	212
Vancomycin	-	292
Ceftriaxone	-	98
Missing	8	7

(c) “In which instances would you treat an asymptomatic bacteriuria with an otherwise stable patient (choose up to 3)?” The preferred answer is “pregnancy,” as the others would not warrant treatment if the patient is not systematically unwell.		
Correct situations for treating asymptomatic bacteriuria		
Situation	Count	
Pregnancy	808	
Urinary catheter	546	
Highly resistant	350	
None of the above	139	
Missing	8	

When asked about preference for broad-spectrum antibiotics, 10.7% (n = 126) strongly agreed and 20.1% (n = 237) agreed that they favored broad-spectrum agents, while 31.3% (n = 369) disagreed and 11.4% (n = 134) strongly disagreed. Finally, consideration of culture results was high: 42.8% (n = 505) strongly agreed and 35.1% (n = 414) agreed that they routinely incorporated culture and sensitivity data when prescribing antibiotics (Supplementary File 3: Table 2). However, 224 (19%) stated that culture results were available and trustworthy, 254 (21.5%) stated that they were available but difficult to obtain, and 416 (35.2%) stated that they were available but expensive (Table 3).

Case-scenario questions assessing clinical practice showed interesting results. In the scenario involving appropriate first-line antibiotic selection for a non-pregnant patient with pyelonephritis who was not overtly septic (for which respondents could indicate only one appropriate first choice), ciprofloxacin (37.5%, n = 442) and ceftriaxone (29.3%, n = 345) were most frequently chosen, followed by nitrofurantoin (12.5%, n = 147), meropenem (11.9%, n = 140), and co-amoxiclav (7.7%, n = 90). Of these, nitrofurantoin is not suitable because of poor penetration into the renal tract, and co-amoxiclav and ceftriaxone do not act against extended-spectrum beta-lactamases, the prevalence of which may vary in different patient cohorts.

For a similar question addressing urinary tract infection without pyelonephritis, responses were less variable: nitrofurantoin (50.4%, n = 594) and ciprofloxacin (27.5%, n = 324) were the most commonly selected agents, followed by amoxicillin/clavulanic acid (10%, n = 117) and ceftriaxone (8.7%, n = 103), whereas 2.4% (n = 28) selected meropenem, an antibiotic not considered a first-line choice in such scenarios (Table 4).

When asked to choose up to three agents that can be effective against *Pseudomonas* species infections, meropenem was most commonly selected (n = 589) (appropriate), followed by ciprofloxacin (n = 381) (appropriate) and trimethoprim-sulfamethoxazole (n = 278) (not appropriate because of intrinsic resistance). Piperacillin-tazobactam received 202 votes (appropriate), amoxicillin-clavulanic acid 78 (not appropriate because of intrinsic resistance), and doxycycline 53 (not appropriate because of intrinsic resistance); eight respondents did not answer. For anaerobic bacterial infections, piperacillin-tazobactam was most frequently selected (n = 444), followed by meropenem (n = 341) and amoxicillin-clavulanic acid (n = 212); 292 participants chose vancomycin and 98 chose ceftriaxone, both of which provide no anaerobic coverage.

Finally, when asked which clinical scenarios justify treating asymptomatic bacteriuria, pregnancy was the most frequently selected indication (68.5%, n = 808) (appropriate), followed by an indwelling urinary catheter (46.3%, n = 546) (not appropriate in the absence of sepsis, as this indicates colonization) and isolation of a highly resistant organism (29.7%, n = 350) (not in itself an indication) (Table 4).

## Discussion

This is the first survey that includes participants across different regions of Syria and the first to capture KAP regarding AMR among physicians and pharmacists. It provides valuable insights into the degree to which respondents considered AMR to be a problem in Syria and globally, their sources of information, their confidence in prescribing antibiotics, and related decision-making. In general, awareness is high; however, we note a reluctance around the use of antibiotic protocols (a core component of AMS), with 26.4% considering them to be a hindrance in clinical practice. Data from this survey will serve as a baseline for introducing relevant interventions.

Regarding knowledge about AMR, we found that respondents across all levels of seniority had a good understanding of its importance and consequences. We also noted high self-reported confidence in prescribing antibiotics, with notable differences by gender and seniority. A recent systematic review and meta-analysis of AMR KAP surveys, including 108 studies and 29,433 health care workers, found that male respondents were more likely than female respondents to have adequate AMR knowledge (59.0% [95% CI 50.5-67.4%] vs 51% [95% CI 40.1-61.9%]) [19].

Although most respondents acknowledged that AMR is a global (89.5%) and national threat (93%), only 66.6% considered it an issue in their hospital. The discrepancy between global and local recognition was also noted in a study from Peru by García *et al.*, where most physicians considered AMR a global (70%) and national concern (65%), but only 22% considered it immediately relevant to their practice [20]. This is something that could influence local AMS programs or prescribing behaviors.

We found confidence in prescribing to be high for initiating therapy (93.1%) (with gender, seniority, and specialty differences) but lower in cases requiring special dosing (e.g., renal or liver failure [40.2%]) or regimen adjustment (45.2%). We emphasize that confidence does not necessarily equate to competence in prescribing. In the review by Jahromi *et al.* [19], it was found that male health care workers were slightly more likely to have adequate AMR knowledge according to their pooled results.

Although attitudes toward AMS were positive (90%,) fewer than half of the respondents considered AMS programs to be effective in their institutions. However, the number of respondents working in facilities with an AMS program in place and the specific program components were unclear, limiting the interpretation of this finding. In a study from Saudi Arabia by Baraka *et al.* [21] involving 184 clinicians, >50% were unaware of AMR programs and 71.2% had no previous AMS experience, suggesting that the implementation of AMS programs needs ongoing support. Weier *et al.* reviewed all AMS programs identified by the ESCMID (European Society of Clinical Microbiology and Infectious Diseases) Study Group on AMS in 2018 and through online searches, identifying 80 programs in total and noting that fewer than half of participants had been aware of one or more available programs. They found that pharmacists (73%) were more aware of AMS programs than physicians (46%), although most programs were aimed at physicians (46%) [22].

The case scenarios demonstrate opportunities to tailor educational and training interventions in Syrian Arabic to the local context (e.g., antibiotic or culture availability). Consistent with this, we noted insufficient opportunities for postgraduate education in AMR or AMS, with more than half of respondents reporting no such training in the past year. To remedy this, nationally adapted education programs on AMR, AMS, and case studies should be implemented from undergraduate through postgraduate levels across different health care worker groups. To remedy gaps in knowledge, further training on infectious disease and clinical microbiology (for both physicians and pharmacists) is required to grow this workforce so that they can support education and AMS programs across specialties such as intensive care and nephrology, as seen in other countries.

### Strengths and limitations

A strength of this work is the large and diverse group of respondents with balanced gender representation, providing a baseline for future interventions. However, the survey timing (spanning the fall of the regime) may have influenced response rates and engagement. Given the mode of dissemination, the sample may have been biased and self-selected, with less engagement from pharmacists, a cadre that is key to AMS implementation. Further research is needed to explore implementation strategies based on this work and guide targeted interventions, such as introducing and enforcing standardized protocols and influencing prescribing practices.

## Conclusion

This study indicates that Syrian health care professionals are motivated regarding AMR and AMS but face under-resourcing in microbiology services and in educational and training opportunities. Interventions tailored to different groups and the local context are needed and will be implemented through university institutions (e.g., University of Aleppo) and relevant ministries (e.g., the Ministry of Higher Education) to support such activities at the national scale as Syria rebuilds its health system.

## Declaration of competing interest

The authors have no competing interests to declare.

## Appendix Supplementary materials

Image, application 1
