# Supplementary material for: A cross-sectional survey of knowledge, attitudes, and practices regarding antimicrobial resistance among Syrian health care professionals
Source: IJID Reg. 2025 Oct 19;17:100789. doi: 10.1016/j.ijregi.2025.100789 (PMC12662071; doi:10.1016/j.ijregi.2025.100789)
Supplement: Supplementary file 1 [file mmc1.pdf]

Part 1. General Questions

Nr	Question	Answer
1	Are you male or female?	Male<br>Female
2	What is your age group?	18-25<br>26-35<br>36-45<br>46-55<br>56-65
3a	Are you a student?	Yes<br>No
3b	If you are a student, what is your degree?	Pharmacy<br>Medicine<br>Nursing<br>Dentistry
4	If you have graduated, what is your degree?	Pharmacy<br>Medicine<br>Nursing<br>Midwifery<br>Dentistry
5	If you have already graduated, how many years have you worked since you graduated?	1 year or less<br>2 years<br>3 years<br>4 years<br>5 years<br>6 years<br>7 years or more
6A	If you are a doctor, what is your speciality?	Medicine /Emergency<br>Surgery<br>Pediatrics<br>Obstetrics and Gynecology<br>Anaesthesia<br>Other
6B	If you are a doctor, what is your current grade?	Resident<br>Attending physician
6C	If you are a doctor, what is your highest degree	MD<br>Masters<br>PhD
7	Do you think knowledge of antibiotic prescribing is important to you now or in the future?	Yes<br>No

8	Do you think antimicrobial resistance is a problem?	It is a problem<br>It is not a problem<br>I do not know
9	Do you think antimicrobial stewardship (correctly prescribing antibiotics at the right time, type and duration) is important?	It is a problem<br>It is not a problem<br>I do not know
10	How often do you prescribe antibiotics?	More than once a day<br>Once a day<br>3-5 times per week<br>1-2 times per week<br>Less than once a week

Part 2: These statements are about your confidence in different aspects of antibiotic prescribing. This will support future educational interventions.

11	I am confident that I know when to start antibiotics.	Very confident<br>Somewhat confident<br>Somewhat unconfident<br>Unconfident
11A	When I start antibiotics, I follow a specific guideline.	Never<br>Sometimes<br>About half the time<br>Most of the time<br>Always<br><br>If used, please specify which guideline you follow:<br><br>____________________
12	I am confident in my ability to choose the correct antibiotic dose and interval for different infections	Never<br>Sometimes<br>About half the time<br>Most of the time<br>Always
13	I am confident in my ability to choose the correct antibiotic dose and interval in kidney or liver failure	Never<br>Sometimes<br>About half the time<br>Most of the time<br>Always

14	I am confident in my ability to change antibiotics if the patient is not responding	Never<br>Sometimes<br>About half the time<br>Most of the time<br>Always
15	I am confident in my ability to decide on the duration of antibiotic treatment	Never<br>Sometimes<br>About half the time<br>Most of the time<br>Always
16	I am confident in interpreting microbiology results and choosing the right antibiotic for the bacteria and patient	Never<br>Sometimes<br>About half the time<br>Most of the time<br>Always
17	If you ask advice from a senior colleague, how often do they change your choice of antibiotic?	Never<br>Sometimes<br>About half the time<br>Most of the time<br>Always
18A	How often do you ask for advice about your choice of antibiotic from a colleague?	Never<br>Sometimes<br>About half the time<br>Most of the time<br>Always
18B	I am able to access advice from diaspora infection or microbiology doctors for advice	Never<br>Sometimes<br>About half the time<br>Most of the time<br>Always

These questions are about education and training on AMR and AMS

19A	During the last year, how many times have you received teaching or training on AMR and AMS?	
	A) As part of the academic activities of your department	0<br>1-3 times<br>4-6 times<br>6-10 times<br>>10 times

19B	B) As part of participation in additional courses or CPD (Continuous Professional Development)	0<br>1-3 times<br>4-6 times<br>6-10 times<br>>10 times
	Which of the following sources of information do you use as part of continuous medical education on antibiotics or when you have a specific question on antibiotics? Please check your rating in terms of its usefulness with 1= being very useful, 3 = useful and 5 = not at all. If you are not familiar with a source, please check that option<br><br>Which of the following sources do you find useful when you have a question on antibiotics? Rate your response based on the usefulness of the resource:<br>1= very useful<br>3 = useful<br>5 = not at all useful	
20A	A) Information from senior colleagues or a specialist	1 2 3 4 5<br>Not used
20B	B) Information from colleagues of the same grade	1 2 3 4 5<br>Not used
20C	C) Uptodate	1 2 3 4 5<br>Not used
20D	D) Sanford Antimicrobial Guide	1 2 3 4 5<br>Not used
20E	E) Local or national guidelines (if available)	1 2 3 4 5<br>Not used
20F	F) Others : please write it out<br><br>--------------------------------------	
21	Do you feel that you have enough sources from which to find the needed information on antibiotics?	Yes<br>No, there should be more sources of information (please specify which sources would be useful):

		....

The following statement/ questions are about your knowledge and practice related to antibiotic prescribing. Tell us how strongly you agree or disagree with the following statements

22	Improving my antimicrobial prescribing would reduce AMR in the hospital/ health facility in which I work	Strongly agree<br>Agree<br>Neutral<br>Disagree<br>Strongly disagree
23	When I prescribe antibiotics, I prefer to use broad spectrum antibiotics as it is safer for the patient.	Strongly agree<br>Agree<br>Neutral<br>Disagree<br>Strongly disagree
24	AMR is less relevant if we have newer antibiotics available	Strongly agree<br>Agree<br>Neutral<br>Disagree<br>Strongly disagree
25	When selecting an empiric antibiotic, which of the following are you most concerned about. Choose three.	Cost<br>Resistance<br>Broad spectrum<br>C. difficile risk<br>Side effects<br>Antibiotic failure
26	When I suspect a patient has a viral infection only, I find it difficult to not give antibiotics if they demand it.	Strongly agree<br>Agree<br>Neutral<br>Disagree<br>Strongly disagree
27	When I suspect a patient has a fever only, I find it difficult to not give antibiotics if they demand it.	Strongly agree<br>Agree<br>Neutral<br>Disagree<br>Strongly disagree

28	For inpatients, I reassess antibiotics after 48-72 of starting them to see if they can be changed.	Strongly agree<br>Agree<br>Neutral<br>Disagree<br>Strongly disagree
29	Antibiotics are overused in the hospital/ health facility I work in	Strongly agree<br>Agree<br>Neutral<br>Disagree<br>Strongly disagree
30	Antibiotics are overused in the community	Strongly agree<br>Agree<br>Neutral<br>Disagree<br>Strongly disagree
31	I believe it is difficult to select the correct antibiotic	Strongly agree<br>Agree<br>Neutral<br>Disagree<br>Strongly disagree
32	Antimicrobial resistance is an important problem in my hospital	Strongly agree<br>Agree<br>Neutral<br>Disagree<br>Strongly disagree
33A	Antimicrobial stewardship (choosing the right antibiotic at the right time for the right patient and stopping the antibiotic when it is not needed) is effective in my hospital	Strongly agree<br>Agree<br>Neutral<br>Disagree<br>Strongly disagree
33B	My hospital/ health facility has a program which monitors and addresses antibiotic overuse	Strongly agree<br>Agree<br>Neutral<br>Disagree<br>Strongly disagree
34	When I decide which antibiotic to use, I am affected by the availability and costs of antibiotics	Strongly agree<br>Agree<br>Neutral<br>Disagree<br>Strongly disagree

35	Patients' attitudes towards antibiotics contribute to overuse of antibiotics	
	a- Among inpatients	Strongly agree<br>Agree<br>Neutral<br>Disagree<br>Strongly disagree
	b- Among outpatients	Strongly agree<br>Agree<br>Neutral<br>Disagree<br>Strongly disagree
36	I take into account microbiology results when I prescribe antibiotics for a patient	Strongly agree<br>Agree<br>Neutral<br>Disagree<br>Strongly disagree
37a	Microbiology results are:	Available and reliable<br>Available but not reliable<br>Available but not easily available<br>Available but too costly for patients<br>Available but take too long to get the result<br>Not available
37b	I communicate directly with the microbiology laboratory to ensure that the samples are collected optimally, sent appropriately, any particular conditions are met and to discuss the results.	Always<br>most often.<br>Often.<br>Sometimes.<br>never
38	Antibiotic restrictions negatively affect patient care because I cannot prescribe the antibiotic that I would like to.<br>If there is no restricted policy, tick 'No restricted policy in my hospital'	Strongly agree<br>Agree<br>Neutral<br>Disagree<br>Strongly disagree<br>No restricted policy in my hospital

39	The development of a local guidelines would be more useful than the international ones	Strongly agree<br>Agree<br>Neutral<br>Disagree<br>Strongly disagree
40	Antibiotic guidelines are an obstacle rather than a help to clinical care.	Strongly agree<br>Agree<br>Neutral<br>Disagree<br>Strongly disagree
41	I would like the provision of more education on antibiotics and prescribing	Strongly agree<br>Agree<br>Neutral<br>Disagree<br>Strongly disagree
42	I am concerned about the quality of some antibiotics available which means they do not work	Strongly agree<br>Agree<br>Neutral<br>Disagree<br>Strongly disagree

Tell us how strongly you agree or disagree with these statements on antimicrobial resistance

43	Antimicrobial resistance is a problem worldwide	Strongly agree<br>Agree<br>Neutral<br>Disagree<br>Strongly disagree
44	Antimicrobial resistance is a problem in my country	Strongly agree<br>Agree<br>Neutral<br>Disagree<br>Strongly disagree
45	Antimicrobial resistance is a problem in my daily practice	Strongly agree<br>Agree<br>Neutral<br>Disagree<br>Strongly disagree

These questions relate to antimicrobial stewardship (choosing the right antibiotic for the right patient at the right time and stopping it at the right time)

46	Antimicrobial stewardship programs reduce the problem of antimicrobial resistance	Strongly agree<br>Agree<br>Neutral<br>Disagree<br>Strongly disagree
47	In general, antimicrobial stewardship programs improve patient care and safety	Strongly agree<br>Agree<br>Neutral<br>Disagree<br>Strongly disagree
48	Antimicrobial resistance is a problem in my daily practice	Strongly agree<br>Agree<br>Neutral<br>Disagree<br>Strongly disagree
49	I have to make difficult decisions on antibiotics because many antibiotics are unavailable	Strongly agree<br>Agree<br>Neutral<br>Disagree<br>Strongly disagree
50	Prescribing antibiotics can cause damage when a patient does not need them	Strongly agree<br>Agree<br>Neutral<br>Disagree<br>Strongly disagree

These questions explore you knowledge and practices related to antibiotic prescribing

51	Your patient is admitted with urinary sepsis caused by E coli; they are not pregnant and the renal function is normal. They have no allergies. **The patient is clinically stable and there is no evidence of pyelonephritis.** It is sensitive to the antibiotic choices provided. Which is your preferred antibiotic (choose one)?	Co-amoxiclav<br>Nitrofurantoin<br>Meropenem<br>Ciprofloxacin<br>Ceftriaxone

52	Your patient is admitted with a burst appendix and was taken to surgery. They have no medical history and have not previously been to hospital. They have no allergies. What is the most appropriate antibiotic choice (choose one)?	Co-amoxiclav<br>Ceftriaxone and metronidazole<br>Meropenem<br>Ciprofloxacin and metronidazole<br>Piperacillin-tazobactam
53	Your patient is admitted with urinary sepsis caused by E coli; they are not pregnant and the renal function is normal. They have no allergies. **The patient is septic with evidence of pyelonephritis**. It is sensitive to the antibiotic choices provided. Which is your preferred antibiotic (choose one)?	Co-amoxiclav<br>Nitrofurantoin<br>Meropenem<br>Ciprofloxacin<br>Ceftriaxone
54	Your patient is admitted with septic shock with an unknown cause. They have no past medical history and have not previously been to hospital. You suspect Gram negative sepsis. You initiate sepsis management including fluid resuscitation. What is the most appropriate antibiotic choice (choose one)?	Co-amoxiclav<br>Nitrofurantoin<br>Meropenem<br>Meropenem and vancomycin<br>Ceftriaxone and amikacin
55	In which situations is it correct to treat asymptomatic bacteriuria? Choose all that apply	if the patient is pregnant<br>if they have a catheter<br>if it very resistant<br>none of the above
56	Which antibiotics can be effective against Pseudomonas? Choose all that apply	Co-amoxiclav<br>Ciprofloxacin<br>Meropenem<br>Doxycycline<br>Bactrim<br>Tazocin
57	Which antibiotics can be effective against MRSA? Choose all that apply	Co-amoxiclav<br>Vancomycin<br>Meropenem<br>Linezolid<br>Clindamycin
58	Which antibiotics have anaerobic activity? Choose all that apply	Co-amoxiclav<br>Piperacillin-tazobactam<br>Meropenem<br>Ciprofloxacin<br>Ceftriaxone<br>Vancomycin

Please add any other comments that you would like to make that are relevant to AMR/ AMS

—---------------

If you would like to receive the results of this survey or be kept informed of the progress or future work, please include your email:

—--------------

Thank you very much for filling this survey

IMPERIAL

Imperial College Research Ethics Committee
Imperial College London
South Kensington Campus
Level 5, Sherfield Building
London
SW7 2AZ
rgitcoordinator@imperial.ac.uk
Tel: +44 (0)207 594 9456

06/06/2024

Dear Alhosni Hisham

Study Title: GMPH: What are the Syrian healthcare professionals' knowledge, attitudes, and practices concerning antimicrobial resistance and stewardship in the different governorates of Syria?

ICREC Reference number: 7060010

The above study was approved by your Head of Department on 05/06/24 and by the Research Governance and Integrity Team (RGIT) on 23/05/24.

Under the Imperial College Research Ethics Committee process, a study that has been reviewed by the Research Governance and Integrity Team (RGIT) and Head of Division/Department, where no significant ethical issues have been identified in the protocol or ethics application, can be granted RGIT approval without requiring it to go to full committee.

Documents
The documents reviewed were:

- Application Form (v1, 06/06/2024)
- Protocol (v1.0,29/04/2024)
- Participant Information Sheet (v1.0, 29/04/2024)
- Survey (v2, 21/05/2024)

Yours sincerely
Ruth Nicholson
Digitally signed by Ruth Nicholson
Date: 2024.06.06 10:30:15 +01'00'
Ruth Nicholson
Head of Research Governance and Integrity
Imperial College London

Supplementary File 3: Figure 1: Confidence in prescribing antibiotics by gender

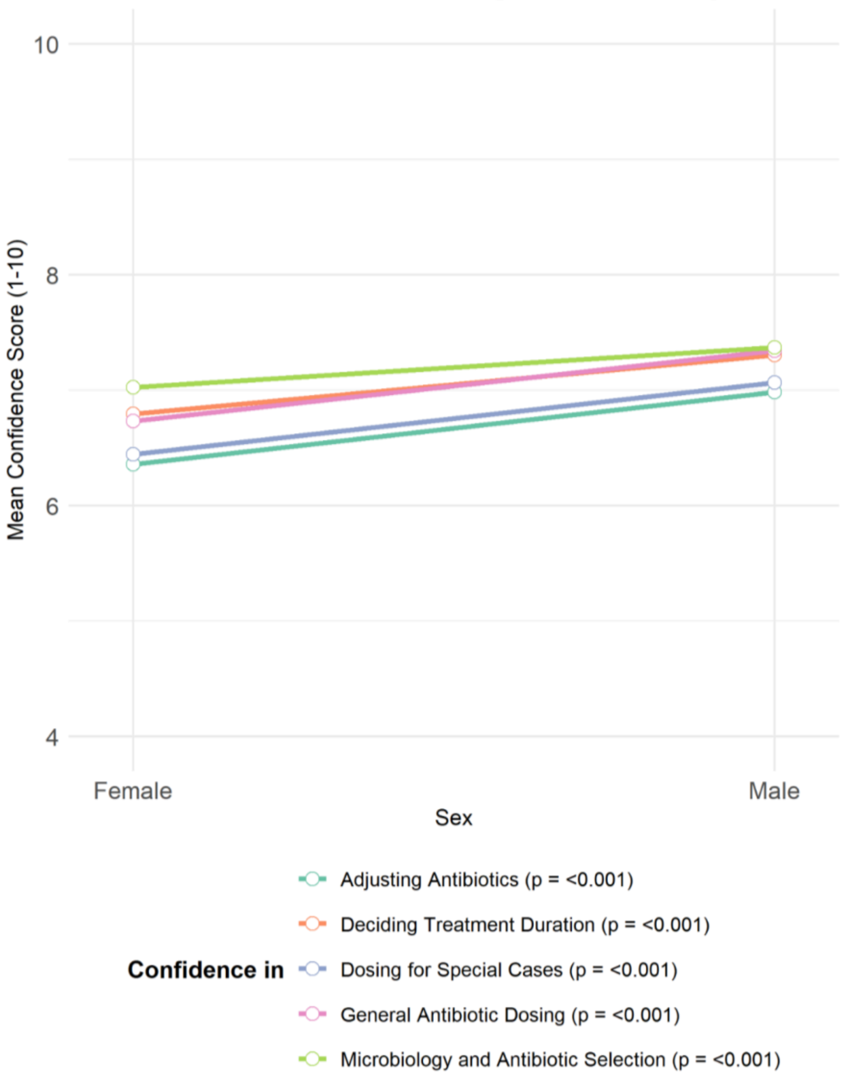

Supplementary File 3: Figure 2: Confidence in prescribing antibiotics by professional category

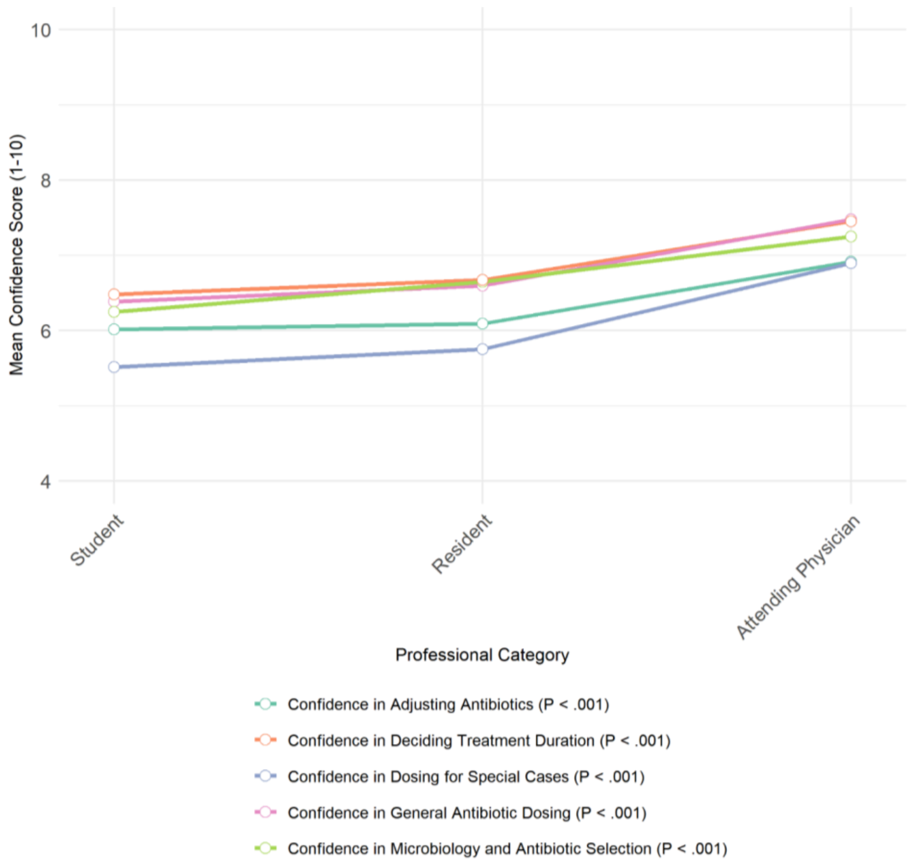

Supplementary File 3: Figure 3: Confidence in Antibiotic Prescription and AMS by Specialty. Note that ANOVA (p-values) could not be generated as some of the categories of speciality were too small.

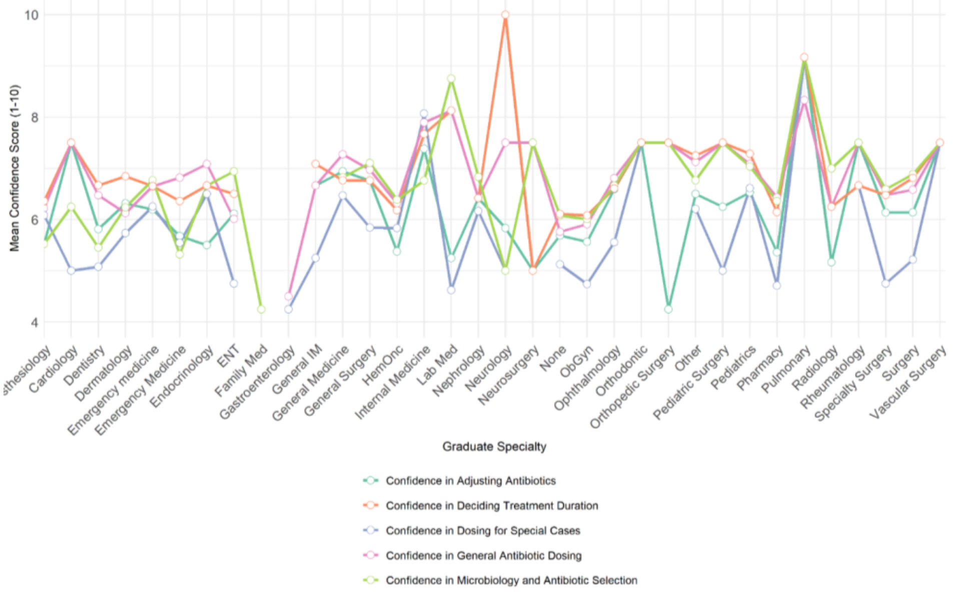

Commented [علا1]: Adding N numbers would be useful

Supplementary File 3: Table 1: AMS Practices. (can go in appendix; decision after paper is finalized).

	Response	N (%)
Frequency of prescribing antibiotics	1-2 times a week	172 (14.6)
	3-5 times a week	249 (21.1)
	less than once a week	158 (13.4)
	more than once daily	469 (39.8)
	once daily	127 (10.8)
	Missing	4 (0.3)
Preference for Broad-Spectrum Antibiotics	Strongly agree	126 (10.7)
	Agree	237 (20.1)
	Neutral	307 (26)
	Disagree	369 (31.3)
	Strongly disagree	134 (11.4)
	Missing	6 (0.5)
Consider Culture Results When Prescribing Antibiotics	Strongly agree	505 (42.8)
	Agree	414 (35.1)
	Neutral	171(14.5)
	Disagree	61 (5.2)
	Strongly disagree	15 (1.3)
	Missing	13 1.1)

Supplementary File 3: Table 2. Factors associated with antibiotic prescribing.

Predictor	OR	95% CI	p-value (<0.05 denotes significance)
Gender:			
Male	1.48	(1.17-1.88)	0.001*
Governorate			
Damascus	1.1	(0.76-1.58)	0.61
Hama	1.64	(0.93-2.89)	0.09
Homs	1.56	(0.96-2.51)	0.071
Idleb	5.23	(3.37-8.1)	<0.001*
Lattakia	0.96	(0.6-1.54)	0.877
Rif Damascus	3.31	(1.83-5.99)	<0.001*
Tartous	2.36	(1.25-4.45)	0.008*
Other	3.15	(1.89-5.24)	<0.001*
Seniority			
Attending Physician	1.26	(0.65-2.43)	0.495
Dentistry, Pharmacy, Nursing	0.83	(0.39-1.75)	0.618
Resident	1.25	(0.68-2.31)	0.473
Speciality			
Emergency Medicine	2.71	(1.47-5.01)	0.001*
Internal Medicine	1.21	(0.52-2.81)	0.665
Obstetrics & Gynaecology	3.97	(1.89-8.34)	<0.001*
Paediatrics	5.36	(2.6-11.06)	<0.001*
General Surgery	4.02	(2.1-7.67)	<0.001*
Other	1.48	(0.82-2.67)	0.196
